# From biaxial tests to cardiac digital twins: a morphomechanics agenda for passive myocardium

**DOI:** 10.3389/fphys.2026.1757669

**Published:** 2026-06-23

**Authors:** Fulufhelo Nemavhola, Thanyani Pandelani

**Affiliations:** 1 Department of Mechanical Engineering, Faculty of Engineering and the Built Environment, Durban, South Africa; 2 Department of Mechanical, Bioresources and Biomedical Engineering, School of Engineering, College of Science, Engineering and Technology, Florida, South Africa

**Keywords:** biaxial testing, cardiac digital twin, constitutive modelling, finite element, morphomechanics, myocardium, ventricular mechanics

## Abstract

Passive myocardial mechanics shape ventricular filling, ventricular-ventricular interaction, and the mechanical environment experienced by cardiac cells. Yet many cardiac finite-element and digital-twin models still estimate passive material behaviour mainly from chamber-level pressure-volume or imaging data, which can reproduce global observables while leaving tissue-scale parameters poorly identifiable. In this Perspective, we define morphomechanics as an operational model-data integration framework in which myocardial structure, tissue-scale stress-strain behaviour, and organ-level function jointly determine constitutive-law selection, parameter priors, and calibration constraints for cardiac digital twins. The term is used to fill a practical gap between conventional multiscale modelling and digital-twin calibration: it specifies how experimentally measured tissue mechanics and quantitative microstructure should constrain organ-scale model parameters. We review insights from planar biaxial testing of ventricular myocardium, including porcine, rat, and sheep datasets that reveal nonlinear elasticity, directional anisotropy, and regional heterogeneity. We then examine how passive myocardial properties are currently estimated in digital-twin workflows and identify where tissue-level evidence remains underused. Finally, we propose an actionable roadmap in which biaxial data inform constitutive-law selection and probabilistic parameter priors; diffusion imaging and histology define local material axes and structural anisotropy; and inverse finite-element calibration incorporates tissue-derived constraints through Bayesian priors, regularization penalties, or staged calibration. By explicitly linking myocardial microstructure, tissue-scale mechanics, and organ-scale simulation, a morphomechanics-driven approach could improve physiological fidelity, parameter identifiability, and translational confidence in cardiac digital twins.

## 1 Introduction

Passive myocardial tissue properties are central determinants of ventricular function. They shape diastolic filling, influence systolic performance through preload, and define the mechanical environment experienced by myocytes, fibroblasts, fibro-adipocytes and the coronary vasculature (Liu and Wang, 2019; Emig et al., 2021). Abnormal passive stiffness and anisotropy are hallmarks of hypertensive heart disease, heart failure with preserved ejection fraction and fibrotic cardiomyopathies, and are increasingly considered therapeutic targets in their own right (Li et al., 2020; Emig et al., 2021).The need for improved mechanical parameterisation has become particularly urgent with the emergence of large-scale digital-twin populations and machine-learning-assisted parameter inference frameworks. As cardiac digital twins move toward clinical deployment, ensuring that model parameters remain physiologically meaningful rather than merely numerically convenient has become a critical challenge. Despite significant progress in cardiac biomechanics modelling, the clinical translation of such models remains limited due to challenges in parameterization, validation, and integration of experimental data (Rodero et al., 2023a).

In this article we focus specifically on passive myocardial mechanics, defined here as the elastic and viscoelastic behaviour of myocardial tissue in the absence of active contractile tension generated by sarcomeres. While active contraction strongly interacts with passive stiffness during the cardiac cycle, the present Perspective concentrates on the passive constitutive behaviour that governs diastolic filling and mechanical preload. The discussion primarily concerns ventricular myocardium, where most biaxial datasets currently exist.

Planar biaxial tensile tests have, for more than four decades, provided a benchmark *in vitro* characterisation of passive myocardium, revealing strongly non-linear, anisotropic and regionally heterogeneous behaviour (Demer and Yin, 1983; Yin et al., 1987; Novak et al., 1994; Dokos et al., 2002; Ghaemi et al., 2009). Early work in canine hearts established fundamental features of the stress–strain response and inspired phenomenological strain-energy functions that remain widely used in finite element (FE) models (Humphrey et al., 1990; Guccione et al., 1991; Costa et al., 2001). More recent studies in pigs, rats and sheep have extended these measurements across multiple ventricular walls and species, often under physiologically relevant loading conditions and temperatures (Nemavhola, 2017a; Nemavhola, 2017b; Ngwangwa et al., 2022; Pandelani et al., 2025).

In parallel, the field has seen rapid growth in cardiac digital twins, computational models that combine patient-specific anatomy, fibre architecture, boundary conditions and haemodynamics to simulate cardiac function and support diagnosis or therapy planning (Chabiniok et al., 2016; Corral-Acero et al., 2020; Sel et al., 2024a). These frameworks typically estimate passive mechanical parameters via inverse FE, fitting model outputs to imaging and pressure–volume data, or increasingly to populations of synthetic or real measurements (Wang et al., 2009; Palit et al., 2018; Qian et al., 2025). However, the choice of passive law and the parameter ranges explored are often only loosely informed by tissue-level experiments (Emig et al., 2021; Rodero et al., 2023b; Lewalle et al., 2025a). Recent advances in computational physiology and multiscale modelling have accelerated the development of cardiovascular digital twins capable of integrating anatomical, physiological, and clinical data into patient-specific predictive models (Sel et al., 2024b).Multiscale computational models increasingly serve as digital twins capable of predicting cardiac function under physiological and pathological conditions (Strocchi et al., 2025).

Cardiac digital twins have achieved substantial progress in reproducing global cardiac function, but their passive mechanical parameters often remain weakly identifiable. Chamber-level observables such as pressure-volume loops, global strain, or regional wall motion contain limited independent information about tissue-scale stiffness, anisotropy, and regional heterogeneity. Consequently, multiple parameter sets may reproduce similar organ-level responses while implying different and sometimes physiologically implausible tissue mechanics. This creates a persistent disconnect between experimental myocardial mechanics and organ-scale computational models.

In this Perspective, we propose multiscale soft-tissue morphomechanics as a conceptual and operational bridge linking biaxial tests and cardiac digital twins. By morphomechanics, we mean the integrated use of myocardial architecture, tissue-scale mechanical measurements, and organ-scale functional data to select constitutive models, define parameter ranges, and constrain patient-specific calibration. This usage is deliberately more specific than the broad term multiscale modelling: it focuses on how experimentally measured structure and mechanics are operationally carried into digital-twin parameterization. We use this framework to (i) summarise what modern biaxial experiments have taught us about passive ventricular mechanics, (ii) analyse where tissue-level knowledge is underused in digital-twin pipelines, and (iii) propose a practical roadmap based on standardized protocols, parameter atlases, benchmark problems, and tissue-constrained model selection.

Recent reviews have highlighted this disconnect (Emig et al., 2021). showed that reported “myocardial stiffness” values vary widely across studies, largely because of differences in measurement protocols, strain windows and modelling assumptions, and called for closer integration between experimental and modelling communities. At the same time, digital-twin surveys emphasise the need for principled model selection and parameterisation to support clinical translation (Corral-Acero et al., 2020; Rodero et al., 2023b; Strocchi et al., 2025; Zou et al., 2025).

In this Perspective, we propose multiscale soft-tissue morphomechanics as the conceptual bridge linking biaxial tests and cardiac digital twins. We use “morphomechanics” to emphasise that myocardial mechanics are inseparable from microstructure and architecture: fibre and sheet organisation, laminar structure, extracellular matrix and regional heterogeneity (Holzapfel and Ogden, 2009; Li et al., 2024). We (i) summarise what modern biaxial experiments have already taught us about passive ventricular morphomechanics, (ii) analyse how digital-twin pipelines typically treat passive mechanics and where tissue-level knowledge is under-utilised, and (iii) propose a morphomechanics-driven roadmap comprising standardised protocols, open parameter atlases, benchmark problems and model-selection guidance for digital-twin applications.

## 2 What planar biaxial tests already tell us about passive myocardium

### 2.1 Classical insights

Classical biaxial experiments established several robust features of passive ventricular myocardium: a compliant “toe region” at low strains followed by rapid stiffening, strong anisotropy with higher stiffness in the fibre direction than the cross-fibre direction, and marked regional heterogeneity across the ventricular wall and along the long axis (Demer and Yin, 1983; Yin et al., 1987; Novak et al., 1994). These findings underpinned phenomenological hyperelastic models—typically of exponential form—that are still in widespread use in FE simulations of the heart (Humphrey et al., 1990; Guccione et al., 1991; Costa et al., 2001; Holzapfel and Ogden, 2009).

The early canine studies by (Demer and Yin, 1983) and (Yin et al., 1987) quantified stress–strain behaviour under simultaneous biaxial loading, demonstrating load-path dependence and revealing the importance of testing along physiological fibre orientations. Subsequent shear and multiaxial tests further enriched this picture by quantifying coupling between normal and shear deformations (Dokos et al., 2002; Schmid et al., 2006; Ghaemi et al., 2009).

### 2.2 Modern regional and cross-species datasets

More recent work has generated larger and more systematic biaxial datasets, often with explicit regional sampling and, in some cases, open data sharing.

In porcine myocardium (Nemavhola, 2017a; Nemavhola, 2017b; Nemavhola, 2021), performed planar biaxial testing on left ventricular (LV), mid-wall/interventricular septal (MDW) and right ventricular (RV) patches from healthy pig hearts at physiological temperature. These studies demonstrated marked non-linearity and anisotropy in all regions, higher stiffness and anisotropy in LV compared to RV, and MDW behaviour intermediate between the two. They also quantified regional differences in the length of the toe region and in strain-energy storage, and reported constitutive parameters for common hyperelastic models together with regional moduli and anisotropy indices.

In rat myocardium, Ngwangwa et al. (2022) characterised passive biaxial properties of LV, septum and RV in Wistar rats under equi-biaxial tension, reporting direction-specific moduli, anisotropy indices and strain-energy densities. Their findings were broadly consistent with the porcine data, with LV and septum stiffer than RV, and RV storing relatively more strain energy at higher deformations (Nemavhola, 2017a; Nemavhola, 2017b; Nemavhola, 2021). further contributed rat datasets and parameter estimation strategies, including preprint-level open data resources. These data complement earlier biventricular finite element models of rat hearts subjected to infarction and RV overload, which combined realistic geometries with structure-based constitutive laws to study regional stress–strain distributions (Masithulela, 2015a; Masithulela, 2015b; Masithulela F., 2016; Masithulela F.J., 2016).

In sheep myocardium (Pandelani et al., 2025), reported passive biaxial properties of LV, mid-wall and RV under physiologically relevant conditions. Patterns were again similar to those seen in pigs and rats, including LV stiffest and most anisotropic, MDW intermediate, and RV most compliant but capable of high strain-energy storage—supporting the mechanical similarity of sheep myocardium to human tissue and reinforcing its role as a large-animal model.

**Figure 1:**
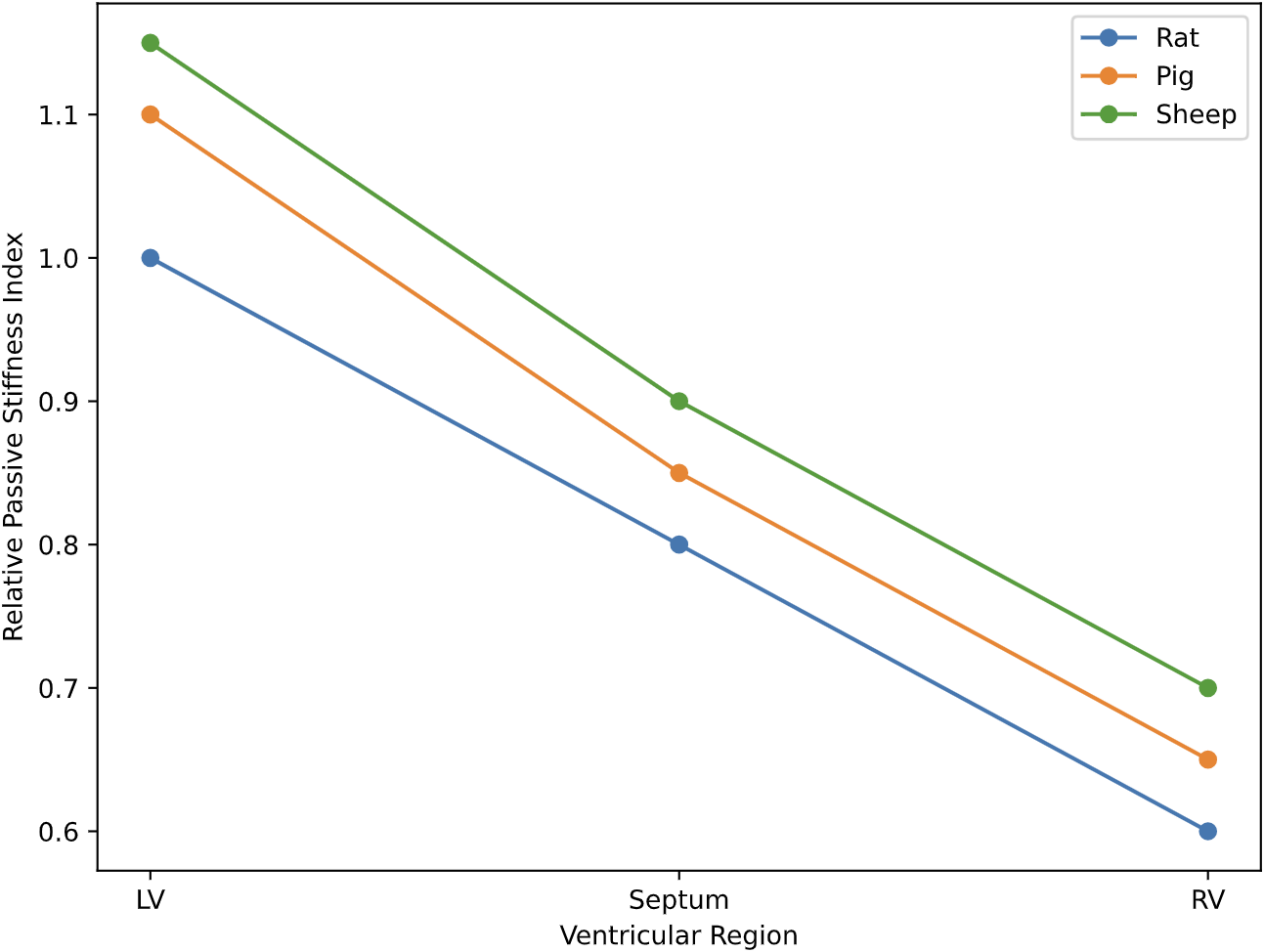
Regional stiffness gradients across ventricular regions in multiple species. Conceptual representation of passive myocardial stiffness gradients observed in experimental studies of ventricular myocardium. Across several mammalian species, including rat, pig, and sheep, the left ventricle (LV) typically exhibits the highest passive stiffness and strongest anisotropy, the interventricular septum shows intermediate mechanical behaviour, and the right ventricle (RV) displays the greatest compliance. These gradients are consistent with the functional roles of the ventricular chambers and are associated with differences in myocardial fibre architecture, collagen density, and laminar organization. Such regional mechanical heterogeneity has important implications for constitutive modelling and should be considered when parameterizing cardiac digital twin models.

Experimental measurements of passive myocardial mechanics consistently reveal regional stiffness gradients across ventricular walls. As illustrated in **Figure 1**, studies across multiple mammalian species show that the left ventricle typically exhibits the highest passive stiffness and anisotropy, followed by the interventricular septum, while the right ventricle remains comparatively compliant. These mechanical differences are believed to reflect underlying structural organization, including fibre alignment, collagen network density, and ventricular loading conditions. Such regional gradients have important implications for computational modelling, because many cardiac digital twin frameworks assume homogeneous passive material properties across the ventricles. Incorporating experimentally observed regional stiffness differences may therefore improve the physiological realism of patient-specific simulations.

Over the past two decades, several experimental studies have characterized the passive mechanical behaviour of ventricular myocardium using planar biaxial testing. These investigations span multiple mammalian species and ventricular regions and collectively provide a growing body of evidence on myocardial anisotropy, nonlinear elasticity, and regional mechanical heterogeneity. Although experimental protocols and species differ, the reported findings reveal broadly consistent patterns in ventricular morphomechanics. In particular, the left ventricle generally exhibits the highest passive stiffness and strongest anisotropy, the interventricular septum or mid-wall demonstrates intermediate mechanical behaviour, and the right ventricle remains comparatively compliant. A summary of representative datasets across species is provided in **Table 1**, highlighting the convergence of experimental observations despite differences in heart size, physiology, and experimental methodology.

**Table 1:** Representative biaxial datasets describing passive myocardial mechanics across species and ventricular regions.

Species	Ventricular regions studied	Mechanical findings	Key references
Rat	LV, Septum, RV	LV stiffer than RV; moderate anisotropy; regional differences in strain energy	Nemavhola, 2017a; Ngwangwa et al., 2022
Pig	LV, Mid-wall, RV	Strong nonlinearity; LV highest stiffness; MDW intermediate; RV most compliant	Nemavhola, 2017a; Nemavhola, 2017b
Sheep	LV, Septum, RV	Similar LV–RV stiffness gradient; consistent anisotropic behaviour	Pandelani et al., 2025
Canine	LV wall regions	Strong fibre-direction stiffness and nonlinear behaviour	Demer and Yin, 1983; Yin et al., 1987
Human (limited data)	LV tissue samples	Passive stiffness varies with disease and fibrosis	Li et al., 2020; Emig et al., 2021

The table summarizes key experimental studies that quantified passive myocardial mechanical properties using planar biaxial testing across several mammalian species. These studies consistently report nonlinear stress–strain behaviour, directional anisotropy between fibre and cross-fibre orientations, and regional mechanical heterogeneity across ventricular walls. Across species, the left ventricle typically exhibits the highest passive stiffness and anisotropy, the interventricular septum or mid-wall shows intermediate behaviour, and the right ventricle remains comparatively compliant. These cross-species patterns suggest that regional mechanical gradients are a robust feature of ventricular morphomechanics and should be considered when parameterizing constitutive models for cardiac digital twin simulations.

Taken together, these datasets suggest three consistent cross-species patterns: LV stiffness exceeds septal or mid-wall stiffness, which in turn exceeds RV stiffness across physiologically relevant strain ranges; anisotropy is strongest in LV, moderate in septum/MDW and weakest in RV; and RV exhibits disproportionate strain-energy storage at large strains, consistent with its role as a volume-accommodating chamber (Nemavhola, 2017a; Ngwangwa et al., 2022; Pandelani et al., 2025). These patterns persist despite substantial differences in heart size, heart rate and global haemodynamics between rats, pigs, sheep and humans (Li et al., 2020; Emig et al., 2021).

### 2.3 Towards spatial morphomechanical mapping

Technical advances are enabling more spatially resolved measurements, combining planar biaxial testing with full-field displacement or thickness measurements and with microstructural characterisation (e.g., histology, diffusion imaging) (Li et al., 2020; Ndlovu et al., 2022). Such methods allow local stiffness and anisotropy to be mapped across tissue patches rather than inferred from a single gauge region, aligning naturally with a morphomechanics perspective: regional variations in fibre architecture, wall thickness and extracellular matrix content can be correlated with local mechanical behaviour (Holzapfel and Ogden, 2009; Emig et al., 2021).

In summary, biaxial experiments already provide a rich but fragmented picture of passive ventricular morphomechanics. The challenge is less a shortage of data than a lack of standardisation, curation and integration with organ-scale models.

### 2.4 What biaxial tests reveal beyond pressure–volume measurements

Pressure–volume measurements provide critical information about global ventricular compliance and chamber-level diastolic function. However, these measurements alone cannot uniquely determine the underlying constitutive behaviour of myocardial tissue. Multiple combinations of stiffness parameters and anisotropy ratios can reproduce the same pressure–volume loop, leading to well-known parameter identifiability problems in inverse finite-element estimation.

Planar biaxial testing provides complementary information by directly measuring tissue-level stress–strain responses along independent loading axes. Such experiments reveal directional stiffness differences between fibre and cross-fibre orientations, quantify anisotropy ratios, and expose regional heterogeneity across ventricular walls. These mechanical features are largely invisible to chamber-level measurements but critically influence local strain distributions in computational models.

At the same time, it is important to recognise the limitations of biaxial experiments. They are typically conducted ex vivo under quasi-static conditions and therefore do not capture active–passive coupling present in the beating heart. In addition, patient-specific datasets remain limited, meaning that current applications rely primarily on animal models or population-level parameter ranges.

### 2.5 Structural origins of regional mechanical differences

Regional mechanical differences across ventricular walls likely reflect underlying structural organisation. The left ventricle contains a denser collagen network and more pronounced fibre alignment compared with the right ventricle, contributing to higher stiffness and anisotropy. In contrast, the right ventricle exhibits greater compliance consistent with its role as a volume-accommodating chamber. These structural features appear broadly conserved across mammalian species, although disease processes such as fibrosis, hypertrophy, or myocardial infarction can substantially alter these relationships.

Quantitative microstructural data enter constitutive models through the definition of local material axes and structural stiffness modifiers. Diffusion-tensor imaging, structure-tensor analysis, or rule-based fibre generation provides local fibre, sheet, and sheet-normal directions. The strain tensor is then projected onto these directions so that the constitutive law contains fibre, cross-fibre, sheet, and shear contributions. Histology can further quantify collagen volume fraction, collagen orientation dispersion, fibrosis burden, and laminar organization. These descriptors may be used to adjust regional stiffness scaling factors, anisotropy coefficients, or dispersion parameters. For example, a region with increased collagen content or reduced fibre dispersion may be assigned a higher prior stiffness or stronger fibre-direction weighting, whereas a region with more compliant RV architecture may be assigned lower stiffness and weaker anisotropy. Thus, DTI and histology are not only descriptive; they define the material coordinate system and provide quantitative modifiers for the constitutive parameters used in the digital twin.

## 3 How cardiac digital twins currently treat passive mechanics

Cardiac digital twins typically combine patient-specific anatomy (from MRI/CT), fibre architecture (rule-based or diffusion-tensor–derived), electrophysiology and haemodynamics within a unified FE or multi-physics framework (Chabiniok et al., 2016; Corral-Acero et al., 2020; Rodero et al., 2023b). Passive mechanics are embedded through a chosen strain-energy function for myocardium and a set of material parameters estimated by fitting model outputs, such as pressure–volume loops, global strains or regional wall motion, to clinical data (Wang et al., 2009; Palit et al., 2018; Li et al., 2024; Qian et al., 2025). Recent developments in high-performance computing have enabled multiphysics digital twin simulations capable of capturing electrophysiology, haemodynamics, and myocardial mechanics within unified frameworks (Viola et al., 2023). Recent inverse finite-element frameworks have been proposed to estimate passive myocardial parameters from imaging-derived deformation data (Shi et al., 2024).

Common features of current practice include the use of exponential transversely isotropic laws (e.g., Guccione-type or structurally based formulations) as default passive models (Humphrey et al., 1990; Guccione et al., 1991; Holzapfel and Ogden, 2009; Göktepe et al., 2010), calibration of a small number of “stiffness scaling factors” per patient via inverse FE, and limited attention to transmural or cross-wall heterogeneity. In many frameworks, LV is treated as mechanically homogeneous, and RV may be modelled using the same law and parameters as LV, despite the consistent regional differences described above (Nemavhola, 2017a; Emig et al., 2021; Ngwangwa et al., 2022). Machine-learning-based finite-element approaches have recently been proposed to represent nonlinear myocardial material behaviour while reducing computational cost (Zhang et al., 2022).

A major challenge in calibrating cardiac digital twin models lies in the limited information content of chamber-level measurements alone. Pressure–volume loops and imaging-derived strain fields provide important insights into ventricular function, but they constrain only a small subset of the parameters governing myocardial constitutive behaviour. As a result, several combinations of stiffness and anisotropy parameters may reproduce similar macroscopic outputs, leading to poorly identifiable inverse modelling problems. Tissue-level experiments provide complementary mechanical information that cannot be inferred from chamber-scale measurements alone. In particular, planar biaxial tests directly measure directional stress–strain responses and therefore quantify anisotropy, nonlinear elasticity, and regional mechanical heterogeneity. The complementary roles of chamber-scale and tissue-scale measurements are summarized in **Table 2**, highlighting how integrating both types of data can improve the robustness and physiological interpretability of cardiac digital twin parameterization.

**Table 2:** Mechanical information obtainable from chamber-level measurements versus tissue-level biaxial experiments in cardiac biomechanics.

Measurement type	Mechanical information provided	Limitations
Pressure–Volume measurements	Global ventricular compliance, chamber stiffness, diastolic filling behaviour	Cannot uniquely determine tissue anisotropy or regional mechanical properties
Imaging-derived strain fields	Regional deformation patterns	Limited ability to infer constitutive parameters
Planar biaxial testing	Directional stress–strain behaviour, anisotropy ratios, strain-energy density	Ex vivo testing; lacks physiological boundary conditions
Combined PV + tissue mechanics	Improved parameter constraints and model calibration	Requires integration across experimental and computational domains

The table compares the types of mechanical information obtained from pressure–volume (PV) measurements, imaging-derived strain data, and planar biaxial tissue experiments. Chamber-level measurements provide important insight into global ventricular compliance and overall diastolic behaviour but offer limited ability to uniquely determine myocardial constitutive parameters. In contrast, biaxial testing directly quantifies tissue-level stress–strain behaviour along multiple loading directions, revealing anisotropy, nonlinear elasticity, and regional mechanical heterogeneity. Combining chamber-scale measurements with tissue-scale experimental data provides complementary constraints that improve parameter identifiability and physiological plausibility when calibrating cardiac digital twin models.

Because organ-scale data provide a limited number of independent constraints, these inverse problems are often ill-posed: many different combinations of stiffness and anisotropy can fit the same pressure–volume loop or global strain field (Remme et al., 2004; Schmid et al., 2006; Palit et al., 2018; Shi et al., 2024). Without informative priors or constraints from tissue-level experiments, parameter estimates may drift into physiologically implausible regimes. From a morphomechanics viewpoint, the consequence is that many digital twins have weakly grounded passive mechanics: they reproduce global function, but their microstructural and meso-scale implications are ambiguous (Rodero et al., 2023b; Lewalle et al., 2025a; Ohnemus et al., 2025). This limits interpretability, especially when asking questions about diastolic dysfunction, regional remodelling or the mechanical impact of devices and surgical interventions.

Recent work on large-scale digital-twin populations and AI-assisted parameter inference has highlighted both the potential and the risks of increased automation (Qian et al., 2025; Tasmurzayev et al., 2025; Ugurlu et al., 2025). Methodologies that do not embed tissue-level knowledge may produce internally consistent but physiologically unrealistic parameter ensembles, undermining clinical confidence.

Several recent studies have begun incorporating regional mechanical heterogeneity or tissue-level measurements into cardiac digital twin frameworks. Examples include inverse modelling approaches that combine MRI-derived strain data with experimentally constrained constitutive laws and population-based parameter estimation strategies.

**Figure 2:**
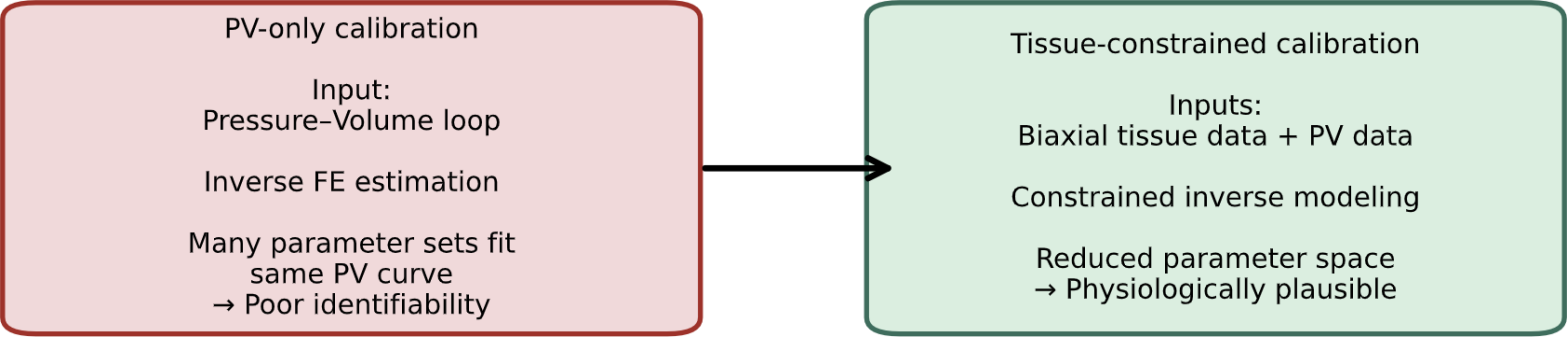
Parameter identifiability in cardiac digital twin calibration: pressure–volume data alone versus tissue-constrained calibration. Illustrates the identifiability problem schematically. Chamber-level pressure-volume and strain measurements constrain global function, but not all combinations of passive stiffness, anisotropy, and regional heterogeneity. Adding biaxial tissue measurements narrows the feasible parameter space and helps ensure that digital-twin calibration remains consistent with experimentally observed myocardial behaviour.

A central challenge in cardiac digital twin calibration is parameter identifiability. Chamber-level observables such as pressure–volume relationships or global strain measurements provide only limited independent constraints on myocardial constitutive parameters. As a result, several parameter combinations may produce similar macroscopic outputs, making the inverse problem ill-posed. **Figure 2** illustrates this challenge schematically. When model calibration relies solely on pressure–volume data, multiple parameter sets can reproduce the same ventricular response. By contrast, incorporating tissue-level measurements such as biaxial stress–strain relationships provides additional constraints that restrict the admissible parameter space. Integrating such experimental information therefore improves the physiological interpretability and robustness of digital twin models.

## 4 Multiscale soft-tissue morphomechanics as a bridge

Operationally, morphomechanics denotes a workflow in which quantitative morphology and mechanics jointly constrain a computational model. In passive myocardium, morphology refers to measurable structural descriptors such as fibre orientation, sheet or laminar organization, wall region, collagen content, fibrosis burden, and orientation dispersion. Mechanics refers to measured stress-stretch behaviour, anisotropy ratios, strain-energy storage, and fitted constitutive parameters obtained from tissue experiments. The morphomechanics step is the explicit translation of these measurements into constitutive-law choice, parameter distributions, spatial heterogeneity, and calibration constraints for organ-scale finite-element models.

This differs from conventional multiscale modelling in emphasis. A multiscale model may connect cell, tissue, and organ representations, but it does not necessarily specify how experimental biaxial measurements or microstructural metrics should restrict patient-specific parameter estimation. Morphomechanics therefore fills a practical gap: it provides rules for keeping digital-twin parameters physiologically anchored while still allowing personalization to patient imaging and haemodynamic data.

A practical example is regional passive parameterization of a biventricular digital twin. Biaxial tests on LV, septal/mid-wall, and RV tissue provide region-specific stress-stretch curves, toe-region behaviour, tangent moduli, strain-energy density, and anisotropy ratios. These data can be fitted to candidate passive laws, such as Guccione-type or structurally based exponential formulations, and the resulting parameter distributions can be used as priors for each ventricular region. Patient-specific pressure-volume and strain data then update these priors rather than replacing them. In this way, the model may personalize a global stiffness scale or regional correction factors while preserving experimentally observed constraints such as LV stiffness greater than septal stiffness greater than RV stiffness, or fibre-direction stiffness exceeding cross-fibre stiffness over the relevant strain range.

We use *multiscale soft-tissue morphomechanics* to describe the quantitative linking of myocardial microstructure and architecture to observed mechanical behaviour across scales. In this view, biaxial tests probe meso-scale morphomechanics of tissue patches (millimetres to centimetres), imaging and histology reveal microstructural and architectural information (fibres, sheets, extracellular matrix) at sub-millimetre scales, and digital twins capture organ-scale behaviour, integrating anatomy, loads and boundary conditions (Holzapfel and Ogden, 2009; Chabiniok et al., 2016; Emig et al., 2021).

A morphomechanics agenda therefore asks how microstructural and architectural information can be linked to constitutive models calibrated on biaxial data; how these models and their parameter ranges can be carried upward into organ-scale digital twins in a principled way; and how disease processes—fibrosis, hypertrophy, remodelling—alter these links over time (Liu and Wang, 2019; Li et al., 2020; Rodero et al., 2023b). Biaxial data are central here: they provide direct experimental access to meso-scale behaviour, particularly anisotropy and cross-wall heterogeneity, and they sit at the natural interface between microstructure and organ-scale simulations (Demer and Yin, 1983; Nemavhola, 2017a; Ngwangwa et al., 2022; Pandelani et al., 2025).

Although the morphomechanics framework is presented conceptually as a bottom-up process linking microstructure, tissue mechanics, and organ-scale models, digital twin workflows typically involve bidirectional information flow. Organ-level observations such as regional strain patterns or pressure–volume relationships may inform refinement of tissue-level parameters through inverse modelling. Similarly, disease progression processes such as fibrosis or hypertrophy may alter the microstructure–mechanics relationship over time. Incorporating such feedback loops will be essential for extending morphomechanics toward predictive models of cardiac growth and remodelling.

Embedding this perspective into digital-twin pipelines implies moving beyond “parameter fitting” towards model–data integration across scales: tissue-level experiments inform priors and constraints; microstructure-informed constitutive laws capture architecture; and organ-scale simulations respect these constraints when matching patient data.

**Figure 3:**
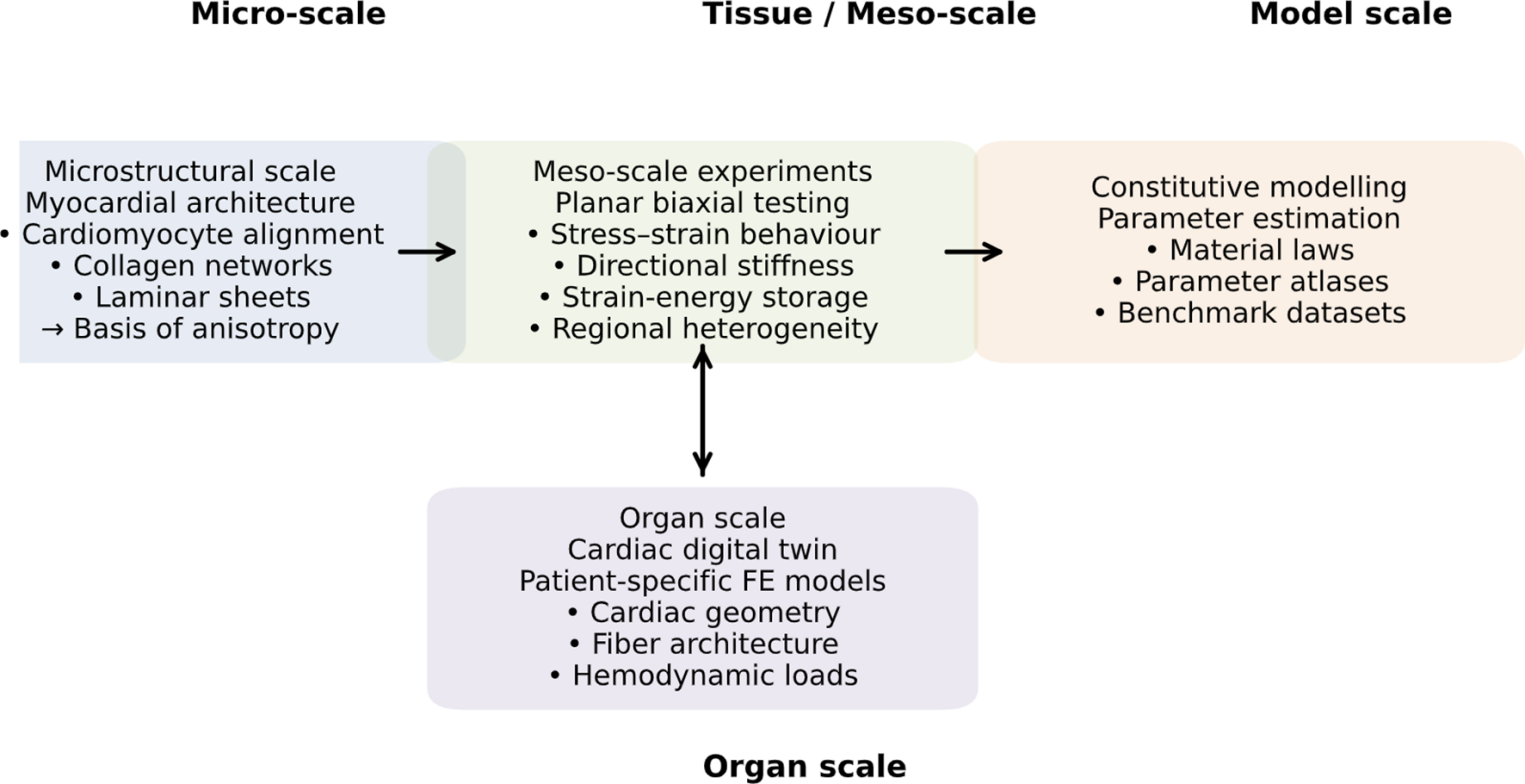
Conceptual architecture of the morphomechanics framework linking myocardial experiments and cardiac digital twins. The morphomechanics framework integrates mechanical measurements and structural information across spatial scales to improve parameterization of cardiac digital twin models. At the microstructural scale, myocardial architecture, including cardiomyocyte alignment, collagen fibre networks, and laminar sheet organization, defines the structural basis of tissue anisotropy. At the meso-scale, planar biaxial experiments quantify passive myocardial stress–strain behaviour and reveal directional stiffness, strain-energy storage, and regional heterogeneity across ventricular walls. These experimental datasets inform constitutive model selection and parameter estimation, forming the basis of curated parameter atlases and benchmark datasets. At the organ scale, these experimentally constrained constitutive laws are embedded in patient-specific finite-element models that combine cardiac geometry, fibre architecture, and hemodynamic boundary conditions to construct cardiac digital twins. The framework also includes bidirectional feedback: organ-level observations from imaging and clinical measurements can refine tissue-level parameter estimation, enabling iterative improvement of model fidelity and physiological interpretability.

The conceptual relationship between myocardial experiments and digital twin modelling is illustrated in **Figure 3**. The morphomechanics framework proposes a structured integration of mechanical and structural information across spatial scales. At the microstructural level, myocardial fibre architecture, collagen networks, and laminar sheet organization define the structural basis of tissue anisotropy and nonlinear elasticity. Planar biaxial testing operates at the meso-scale, providing experimentally measured stress–strain relationships that quantify anisotropy, regional stiffness differences, and strain-energy storage. These tissue-level measurements constrain constitutive models and inform the development of parameter atlases that can be incorporated into organ-scale computational models. At the organ level, patient-specific finite-element simulations integrate anatomical imaging, fibre orientation fields, and physiological boundary conditions to construct cardiac digital twins. Importantly, the framework allows bidirectional interaction across scales: organ-level observations obtained from imaging or clinical measurements can refine tissue-level parameter estimation, while experimentally derived mechanical properties provide physiologically grounded constraints for inverse modelling. This multiscale integration is central to the morphomechanics agenda, enabling cardiac digital twins to move beyond purely phenomenological parameter fitting toward experimentally anchored representations of myocardial mechanics.

## 5 A morphomechanics roadmap: from biaxial data to digital twins

Although previous studies have called for improved standardisation of myocardial mechanical testing, the present roadmap extends these proposals by explicitly linking experimental datasets to digital twin parameterisation pipelines. In particular, we propose combining standardised protocols with parameter atlases, benchmark problems, and modelling guidelines, thereby creating an integrated ecosystem connecting experimental biomechanics with computational physiology. The digital twin paradigm relies on continuous integration of experimental and clinical data streams to update model states and parameters dynamically (Lewalle et al., 2025b).

Biaxial testing also has intrinsic limitations. Tissue patches are removed from their physiological boundary conditions and therefore do not experience ventricular pressure gradients, residual stresses, or transmural load transfer. Furthermore, the interaction between cardiomyocytes and extracellular matrix structures occurs across multiple spatial scales that cannot be fully captured in planar experiments.

### 5.1 Standardise biaxial protocols and reporting

Building on (Emig et al., 2021) recommendations for measuring passive properties and on recent multi-region datasets (Nemavhola, 2017a; Ngwangwa et al., 2022; Pandelani et al., 2025), future myocardium biaxial studies could converge on:

- Harmonised strain windows (e.g., diastolic-range Green–Lagrange strains) with explicit reporting of toe-region and “near-linear” moduli.
- Clear specification of stress and strain measures (Cauchy vs. 2nd Piola–Kirchhoff; engineering vs. Green–Lagrange).
- Minimal metadata sets including species, age, chamber, wall, transmural position, thickness, preconditioning protocol, loading paths and bath conditions (Dokos et al., 2002; Ghaemi et al., 2009; Emig et al., 2021).
- Open sharing of raw data (forces, displacements or stresses and stretches) where ethically and practically feasible (Nemavhola, 2021; Ngwangwa et al., 2022; Pandelani et al., 2025).

Such standardisation would make it far easier to assemble cross-study atlases and to use tissue-level data as priors or constraints in inverse FE workflows.

### 5.2 Build open atlases of passive myocardial parameters

The porcine, rat and sheep studies already provide regional stiffness, anisotropy and, in some cases, fitted hyperelastic parameters (Nemavhola, 2017a; Ngwangwa et al., 2022; Pandelani et al., 2025). Earlier work in canine and human myocardium, compiled in recent reviews, extends this to additional species and conditions (Demer and Yin, 1983; Yin et al., 1987; Li et al., 2020; Emig et al., 2021).

An open “myocardial morphomechanics atlas” could aggregate representative stress–strain curves, moduli, anisotropy indices and strain-energy measures for each species and wall; offer example parameter sets for commonly used strain-energy functions (Humphrey et al., 1990; Guccione et al., 1991; Holzapfel and Ogden, 2009; Göktepe et al., 2010); and provide recommended prior ranges for use in Bayesian or regularised inverse FE estimation in digital-twin pipelines (Palit et al., 2018; Shi et al., 2024; Qian et al., 2025).

It is important to recognise that parameters obtained from biaxial experiments are themselves subject to uncertainty arising from protocol differences, specimen variability, and model non-uniqueness. Several strain-energy formulations may fit the same dataset equally well, leading to parameter degeneracy. The proposed atlas framework should therefore represent parameter ranges probabilistically rather than as single deterministic values. Such probabilistic priors can be incorporated into Bayesian or regularised inverse modelling approaches, improving robustness and reducing the risk of physiologically implausible parameter estimates.

The proposed role of ex vivo biaxial data is not to transfer animal-derived parameters directly into a human digital twin. Instead, these data should be used at four levels. First, they guide constitutive-law selection by identifying whether a model can reproduce the measured toe region, nonlinear stiffening, anisotropy, and regional heterogeneity. Second, they define physiologically plausible priors or admissible ranges for passive parameters, including regional ratios between LV, septum, and RV. Third, they provide reference parameter sets that can be scaled during patient-specific calibration using *in vivo* pressure-volume, imaging-derived strain, and haemodynamic measurements. Fourth, they provide plausibility checks: a personalized solution that fits clinical data but violates well-established tissue-scale behaviour should be treated as under-constrained or physiologically suspect. This bridge must account for species, age, disease state, residual stress, temperature, and the absence of active tone in ex vivo tests; for this reason, atlas values should be represented probabilistically and updated using patient-specific data rather than used as fixed constants.

### 5.3 Develop benchmark problems and datasets for passive myocardial FE

Inspired by benchmark initiatives in other areas of biomechanics and computational physiology (Chabiniok et al., 2016; Rodero et al., 2023b; Lewalle et al., 2025a), the community could define a set of benchmark tests using public biaxial datasets:

- A planar myocardial patch subject to equi-biaxial and non-proportional loading, calibrated against experiments (Nemavhola, 2017a; Ngwangwa et al., 2022).
- A truncated LV wedge under internal pressure, with boundary conditions defined from imaging data and tissue parameters constrained by the atlas (Costa et al., 2001; Wang et al., 2009; Palit et al., 2018).
- A simplified biventricular geometry with prescribed boundary conditions and regionally varying material properties based on LV/MDW/RV differences (Nemavhola, 2017a; Pandelani et al., 2025).

For each benchmark, target curves derived from biaxial data and/or organ-scale measurements would be provided, and modellers could test different constitutive laws and numerical schemes. Cardiac digital-twin frameworks would be expected to demonstrate that their passive models pass a subset of these benchmarks before clinical application (Rodero et al., 2023b; Ohnemus et al., 2025).

### 5.4 Provide model-selection guidance for digital-twin applications

Different digital-twin use-cases place different demands on passive mechanics. Relatively low-parameter models with homogenised stiffness may capture global indices such as ejection fraction and global diastolic stiffness (Remme et al., 2004; Wang et al., 2009). Device design, regional remodelling or surgical planning may require more detailed transmural and regional heterogeneity and accurate strain-energy distributions (Costa et al., 2001; Rodero et al., 2023b; Strocchi et al., 2025).

A morphomechanics perspective suggests that model choice should be explicit and tied to available tissue data. For each application type, the community could define “good practice” recommendations: suitable model families; appropriate parameter ranges from the atlas; and benchmark tests that should be passed. Emerging work on scalable digital-twin populations and AI-based surrogates (Qian et al., 2025; Tasmurzayev et al., 2025; Ugurlu et al., 2025) could then be built on this foundation, rather than learning purely from organ-scale outputs.

Translating tissue-level mechanical knowledge into organ-scale computational models requires a structured integration of experimental, structural, and computational data sources. The morphomechanics framework proposed in this Perspective emphasizes such integration by linking microstructural observations, tissue-scale mechanical measurements, and patient-specific digital twin simulations within a unified modelling workflow. In this approach, experimental datasets derived from biaxial testing inform the calibration of constitutive laws and the development of parameter atlases that constrain inverse modelling procedures. These experimentally grounded parameter ranges can then be incorporated into patient-specific finite-element models constructed from imaging-derived geometries and physiological boundary conditions. The conceptual steps involved in this integration are summarized in **Table 3**, which illustrates how information flows across spatial scales—from myocardial microstructure to organ-scale digital twin simulations—within the proposed morphomechanics framework.

**Table 3:** Proposed morphomechanics integration pipeline linking myocardial experiments with cardiac digital twin modelling.

Step	Input data	Model action	Output for digital twin
1. Tissue-data harmonization	Biaxial stresses, stretches, moduli, strain-energy data	Convert to common stress/strain measures and fit candidate passive laws	Comparable tissue parameter distributions
2. Microstructure mapping	DTI, histology, rule-based fibre fields	Define local fibre/sheet axes and quantify collagen, fibrosis, or dispersion descriptors	Spatial material coordinate system and structural anisotropy modifiers
3. Prior construction	Regional tissue parameters from LV, septum/MDW, RV and disease groups	Build probabilistic priors or admissible parameter ranges for each region	Physiologically plausible parameter space
4. Patient model setup	MRI/CT geometry, wall thickness, fibre architecture, haemodynamics	Construct finite-element model and apply boundary conditions	Patient-specific organ-scale model
5. Tissue-constrained calibration	PV loops, imaging strain, tissue priors	Use Bayesian updating, penalty functions, or staged inverse FE calibration	Personalized parameters that fit clinical data while respecting tissue mechanics
6. Plausibility and uncertainty checks	Posterior parameter sets, predicted strain/stress fields	Check regional stiffness ordering, anisotropy ratios, strain-energy distributions, and prediction intervals	Validated and interpretable cardiac digital twin

The table outlines a conceptual workflow for integrating experimental biomechanics, structural characterization, and computational modelling within a morphomechanics framework. The pipeline progresses from microstructural characterization of myocardial architecture, through tissue-level mechanical testing and constitutive model calibration, to organ-scale digital twin simulations informed by patient-specific imaging and physiological measurements. Each stage provides complementary information that constrains model parameterization and improves physiological interpretability. By integrating experimental datasets, parameter atlases, and computational benchmarks, the morphomechanics pipeline aims to establish a systematic pathway for incorporating tissue-level mechanical evidence into cardiac digital twin development and validation.

### 5.5 Operational pathway for tissue-constrained calibration

A morphomechanics-driven digital-twin workflow can be implemented in five steps. First, tissue-level datasets are harmonized by converting stress and strain measures to common definitions, identifying comparable diastolic strain windows, and extracting moduli, anisotropy ratios, strain-energy densities, and fitted constitutive parameters. Second, candidate constitutive laws are screened against these tissue data; laws that cannot reproduce the measured nonlinear anisotropic response are excluded or used only for applications where such detail is unnecessary. Third, the fitted tissue parameters are converted into regional probabilistic priors or regularization targets, for example separate distributions for LV, septal/mid-wall, and RV myocardium. Fourth, patient-specific inverse finite-element calibration is performed using clinical data while respecting these priors. This may be implemented in a Bayesian framework, where tissue data define the prior probability of parameter sets, or in a deterministic optimization framework, where the objective function combines clinical-data misfit with a penalty for deviation from tissue-derived parameter ranges. Fifth, posterior or optimized parameter sets are validated not only against pressure-volume or strain data, but also against tissue-scale plausibility criteria such as regional stiffness ordering, anisotropy ratios, and strain-energy distributions.

A staged calibration strategy may further improve identifiability. Geometry, wall thickness, cavity volume, and boundary conditions should be established first from imaging and haemodynamic measurements. A global passive stiffness scale can then be calibrated to pressure-volume data. Regional stiffness and anisotropy parameters can subsequently be adjusted using imaging-derived strain fields while constrained by tissue-derived priors. Finally, uncertainty should be propagated to model predictions so that clinically relevant outputs are reported with confidence intervals rather than as single deterministic values.

## 6 Outlook and conclusions

### 6.1 Translational implications of improved passive mechanics

Improved representation of passive myocardial mechanics could influence several clinically relevant applications. In post-myocardial infarction remodelling, regional stiffness changes associated with fibrosis or scar formation alter stress distributions and may influence ventricular dilation and failure progression. Digital twin models incorporating physiologically constrained passive mechanics could therefore improve prediction of remodelling trajectories.

Similarly, accurate representation of myocardial compliance is important for evaluating device interventions such as ventricular assist devices, annuloplasty rings, or ventricular restraint devices. Passive mechanical properties influence how such devices redistribute myocardial stresses and modify ventricular geometry. Finally, improved modelling of passive stiffness may enhance diagnostic assessment of conditions such as heart failure with preserved ejection fraction (HFpEF), where diastolic mechanics play a central role.

Although this Perspective focuses on passive myocardium, the morphomechanics philosophy can also be extended to active myocardial properties. Active tension is generated along structurally defined fibre directions and is influenced by sarcomere organization, calcium handling, activation timing, perfusion, and pathological remodelling. The same model-data logic can therefore be applied: microstructural and electrophysiological data define local activation and contraction axes; experimental or clinical measurements constrain active-tension parameters such as peak active stress, calcium sensitivity, relaxation time, and regional contractility; and organ-scale pressure, volume, and strain data update these parameters during digital-twin calibration. Importantly, passive stiffness and active contractility should not be inferred independently from the same limited global observables without identifiability safeguards. Sequential calibration, joint Bayesian inference, or regularized multi-physics calibration may help avoid conflating increased passive stiffness with reduced active force generation. Extending morphomechanics to active properties would therefore support more complete electromechanical digital twins while preserving the central principle that parameters should remain anchored to measurable tissue structure and function.

### 6.2 Conclusion

Biaxial testing of passive myocardium has generated a substantial body of evidence on ventricular morphomechanics: non-linearity, anisotropy and regional heterogeneity across species (Demer and Yin, 1983; Yin et al., 1987; Novak et al., 1994; Nemavhola, 2017a; Ngwangwa et al., 2022; Pandelani et al., 2025). Cardiac digital twins have, in parallel, become a powerful paradigm for integrating imaging, physiology and modelling in cardiovascular medicine (Chabiniok et al., 2016; Corral-Acero et al., 2020; Rodero et al., 2023b; Sel et al., 2024a). Yet the link between these two domains remains underdeveloped: passive laws and parameters in many digital twins are still chosen pragmatically, with limited anchoring to tissue-level experiments (Emig et al., 2021; Lewalle et al., 2025a; Ohnemus et al., 2025).

We have argued that multiscale soft-tissue morphomechanics provides a natural conceptual bridge and have outlined a practical roadmap for its implementation: standardised protocols and reporting, open parameter atlases, benchmark problems, and model-selection guidance. None of these steps requires fundamentally new technology; they require coordination and shared recognition that tissue-level mechanics are foundational, not optional, in credible digital twins of the heart.

The key message is that cardiac physiology, experimental biomechanics and computational modelling must be treated as a single, coupled enterprise. Biaxial tests, far from being an esoteric laboratory technique, should influence how we parameterise, select and validate the passive myocardium laws at the core of digital-twin models. Implementing this agenda would improve not only the scientific robustness but also the translational potential of next-generation cardiac digital twins.

## Data Availability

The original contributions presented in the study are included in the article/Supplementary Material. Further inquiries can be directed to the corresponding authors.
